# Peculiar Genes Selection: A new features selection method to improve classification performances in imbalanced data sets

**DOI:** 10.1371/journal.pone.0177475

**Published:** 2017-08-14

**Authors:** Federica Martina, Marco Beccuti, Gianfranco Balbo, Francesca Cordero

**Affiliations:** 1 Computer Science Department, University of Turin, Turin, Italy; 2 GSK Vaccines, Siena, Italy; Harbin Institute of Technology Shenzhen Graduate School, CHINA

## Abstract

High-Throughput technologies provide genomic and trascriptomic data that are suitable for biomarker detection for classification purposes. However, the high dimension of the output of such technologies and the characteristics of the data sets analysed represent an issue for the classification task. Here we present a new feature selection method based on three steps to detect class-specific biomarkers in case of high-dimensional data sets. The first step detects the differentially expressed genes according to the experimental conditions tested in the experimental design, the second step filters out the features with low discriminative power and the third step detects the class-specific features and defines the final biomarker as the union of the class-specific features.

The proposed procedure is tested on two microarray datasets, one characterized by a strong imbalance between the size of classes and the other one where the size of classes is perfectly balanced. We show that, using the proposed feature selection procedure, the classification performances of a Support Vector Machine on the imbalanced data set reach a 82% whereas other methods do not exceed 73%. Furthermore, in case of perfectly balanced dataset, the classification performances are comparable with other methods. Finally, the Gene Ontology enrichments performed on the signatures selected with the proposed pipeline, confirm the biological relevance of our methodology. The download of the package with the implementation of Peculiar Genes Selection, ‘PGS’, is available for R users at: http://github.com/mbeccuti/PGS.

## Introduction

High Throughput (HT) experiments have become one of the major source of genomic and trascriptomic information, providing insights into the modulation of gene expression profiles of samples under different conditions. The high potential of HT technologies lies in the quantity of information obtained by one experiment which may increase the possibility of discovering unknown mechanisms underpinning the differences in the biological conditions of interest. For this reason, one of the goals of HT data analysis is the detection of biomarkers for *classification* purposes [1–3]. Despite this high potential, both the high-dimensional output of such technologies and the characteristics of the data set analyzed may represent an issue for classification.

The problem of high-dimensionality of the output is known as the *large p small n problem*, where *p* indicates the number of available predictors—i.e. the thousands of genes assessed—and *n* indicates the number of conditions tested in the experiment—i.e. the number of samples. In the last years, several machine-learning approaches have been proposed to deal with the risk of overfitting deriving from the large p small n problem, leading to a vaste literature about classification methods and features selection/extraction [4–8].

Features selection and features extraction methods reduce the data dimensionality by removing noise and non-informative data and by storing the information needed for the classification purpose in a subset of features called *signature*.

The difference of the two approaches relies in how the signature is created from original data. Indeed, *feature selection* methods shrink the information related to sample classification in the (sub-)optimal subset by removing irrelevant or redundant variables without altering the original representation of the features. *Feature extraction* methods creates new predictors as combination of the features [9].

Once the signature is selected, a classification method is applied to test it. Even though both feature extraction and feature selection are good solutions for the large p small n problem, feature selection allows extraction of meaningful biological rules from the classifier without altering the original characteristics of the selected features and it is less computationally expensive to perform [9].

Another important aspect to consider in classification tasks is the possibility that the data set analyzed is characterized by imbalance among the size of classes. The issue of class imbalance and how to derive good biomarkers from such datasets has received a great deal of interest from the research community and has been the focus of several recent studies [10–13]. Recent works proposed an undersampling-based approach to handle the imbalance between classes size [2, 3]. Also new classifiers, specific for imblanced data sets, were developed and succesfully tested implemented [13, 14]. Classification algorithms are indeed affected in their accuracy performances by imbalance because it is harder to detect the discriminating characteristics of the underrepresented class. This fact leads to high levels of misclassification of samples belonging to the underrepresented class even if there is a good overall accuracy.

The imbalance problem frequently occurs in the new discipline of systems vaccinology [15, 16] where HT experiments are mainly used for the detection of gene signatures predictive of possible adverse reactions related to vaccination or suboptimal vaccine responsiveness. In this clinical context the imbalance in the data sets is due to the fact that a vaccine reaching the human testing phase is supposed to elicit an high response in the majority of the subjects, leading to a poorly populated non-responders response category.

HT studies are commonly used also in cancer-related researches where the capability to distinguish between cancerous and noncancerous tissues or to classify different type of cancer is indeed an inestimable help in medical and biological domain.

Microarrays are a commonly used HT technology which measure the expression levels of large numbers of genes simultaneously or to genotype multiple regions of a genome extracted from a relatively small human samples. The necessity of tools to extract significant information from microarray experiments leads to a vaste amount of softwares and packages available [17–19]. However, the absence of the possibility, in case of imbalanced data sets, to set the parameters of a classifier in order to correctly classify samples belonging to the underrepresented class, is the cause of one of the shortcoming of currently available tools for microarray data analysis.

In this study we present a new features selection method, called *Peculiar Genes Selection* (PGS), to identify predictive biomarkers that are robust to class imbalance and improve the Support Vector Machine (SVM) classification performances in case of imbalanced data sets. The PGS procedure is also applied to a balanced dataset to confirm that it perform well also when the size of the classes is the same. The biomarkers generated by this procedures are therefore a suitable compromise between maximum overall accuracy and correct classification within the underrepresented class.

The novelty of PGS relies on the simple and fast computation of a binary matrix, created by fitting a logistic regression model using single gene expression as predictor of the class label of samples. Grids of parameters for the SVM (i.e. different kernels, same kernels with different initial coefficient and degree, cost and class weights) were explored to maximize one of the three metrics related to classification tasks: overall accuracy, specificity and sensitivity. Since these three metrics are strongly inversely related, the parameters maximizing one do not maximize the other two.

We then compare the classification results obtained on two public microarray data sets with those obtained using one of the available packages for microarray data analysis: the Classification for MicroArrays package, CMA, available under the Bioconductor distribution for R software [18] and with those obtained using MRMD, Maximum Relevance Minimum Distance, a correlation-based feature selection procedure minimizing the feature redundancy and maximizing the correlation with the target class [20].

## Materials and methods

### Data sets

We applied our pipeline on two public sets of data available at NCBI GEO database: a vaccination-related, (GEO accession code: GSE48024) [21], and a cancer-related, (GEO accession code: GSE19804) [22].

#### Vaccination dataset description

This dataset is the result of two studies conducted on two different cohorts of patients. The first cohort, consisting of 119 adult male subjects vaccinated with the 2008–2009 inactivated trivalent influenza vaccine (A/ Brisbane/ 59/ 2007 [H1N1], A / Brisbane/10/ 2007 [H3N2], B/ Florida/ 4/ 2006, Sanofi-Pasteur, Lyon, France). A second cohort, included 128 adult females that received the 2009–2010 trivalent influenza vaccine (A/ Brisbane/ 59/ 2007 [H1N1], A/ Brisbane/ 10/ 2007 [H3N2], B/ Brisbane/ 60/ 2008 strains, Sanofi-Pasteur, Lyon, France).

**Fig 1:**
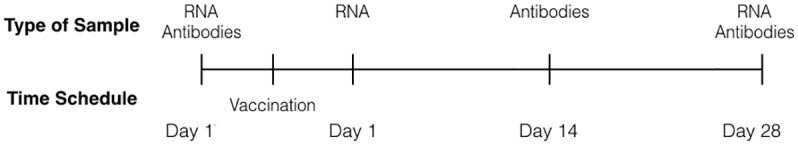
Schematic representation of the vaccination time scheduling experiment used in the present study.

Fig 1 shows the schedule of the visits along with the data that were used for this study. Peripheral blood samples were taken immediately before (day 1^−^) and after vaccination on days 1, 3, and 14 whereas the antibody titers measurements were available before vaccination and on days 14 and 28 after vaccination.

#### Classification of subjects

Since the proposed pipeline is specific for binary classification problems, the selected subjects were classified into two classes: ‘High responders’ and ‘Low responders’. The classification is usually based on the 4 fold-increase of the antibody titers following the vaccination. However, because of the well known baseline effect [23, 24], consisting in an inverse relation between fold-increase and baseline levels of the antibody titers, in influenza-related trials this rough criterion might lead to misclassification. To circumvent this issue, a threshold T_i_, i ∈ { H1N1, H3N2, FluB } was detected as the highest baseline level at which a subject was able to reach a 4 fold-increase in his antibody titers.

#### Cancer data set description

The cancer-related data set consists in 60 paired samples of tumor and adjacent normal lung tissue coming from a study conducted in Taiwan on non-smokers females aged 50 to 70 years old. Three tumors are represented in the data set: Adenocarcinoma, Bronchioloalveolar and Squamous carcinoma (56, 3, 1 sample respectively).

### Methodology

The proposed methodology is a new feature selection procedure called Peculiar Genes Selection (PGS), that detects genes characterizing the class of the samples they belong to. The classification accuracy performances of the gene signature derived from the application of PGS are evaluated using a SVM classifier exploring grids of parameters to maximize the desired metric: overall accuracy and specificity.

#### The Peculiar Genes Selection procedure

In the proposed pipeline the feature selection is performed in three steps:

1. Identification of Differentially Expressed Genes (DEGs)
2. Identification of good predictors
3. Selection of the peculiar good predictors for each class

**Step 1** allows the detection of J differentially expressed genes under two conditions of interest leading to a significative data dimensionality reduction [25].

**Step 2** is based on the computation of a regression model in which single variable levels are used to predict the class label of each subject [26].

Let N be the number of subjects with *n* + *m* = N, *n* being the number of subjects belonging to class 0 (‘C0’) and *m* the number of subjects belonging to class 1(‘C1’). Let also J be the number of DEGs detected in step 1. We indicate with *X*_*j*_ = {*x*_*j*1_, …, *x*_*jN*_}, *j* ∈ {1, …, *J*} the expression of the j-th DEG across all subjects and with $\bar{y}$ the ordered N dimensional vector of true classification labels, where ${\left\{{\bar{y}}_{i}\right\}}_{i=1}^{n}=0$, and ${\left\{{\bar{y}}_{i}\right\}}_{i=n+1}^{N}=1$.

PGS computes J logistic regressions to predict the probability of each subject to be a success, in other words to belong to class ‘1’, given the expression of the j-th independent variable *X*_*j*_. Eq (1) shows the model of logistic regression used, where *p*_*i*_ is the predicted probability of success for subject *i*, *β*_0_ the intercept of the model, *β*_*j*_ the fitted parameter and *X*_*ji*_ the expression of the j-th gene of subject *i*.

$$\begin{array}{c} logit\left(p_{i}\right)=ln(\frac{p_{i}}{1-p_{i}})=\beta_{0}+\beta_{j}X_{ji} \end{array}$$ (1)

The logistic-regression fit lead to J N-dimensional vectors **p** of predicted probabilities of ‘success’, where each component is the *p*_*i*_ calculated in Eq (1). Since the possible class labels are only 0 and 1, the classification vector $\hat{y}$, predicted using the j-th gene as independent variable, is obtained by applying the following criterion:

$$\begin{array}{c} {\hat{y}}_{i}=\{\begin{array}{c} 1\;\text{if}\;p_{i}\geq \tau \\ 0\;\text{else} \end{array} \end{array}$$ (2)

where *τ* stands for pre-selected threshold value that can varies in case of strong imbalance between classes size.

The comparison of $\hat{y}$ with $\bar{y}$ measures the ability of each predictor to correctly classify the subjects. This quantity is called *predictive power* (*pp*) and it is defined as follows:

$$\begin{array}{c} pp_{j}=\frac{\sum_{i=1}^{N}1_{\left\{{\hat{y}}_{i}={\bar{y}}_{i}\right\}}}{N},\;\forall \text{j}\in \left\{1,\ldots ,J\right\}. \end{array}$$ (3)

The J values of *pp* form a distribution of predictive power values, describing the ability of the DEGs of classifying the samples. The *P*, with *P* ≤ *J*, *good predictors* are chosen among the DEGs which *pp* >= *q*, with *q* being a quantile of the predictive powers distribution, describing the minimal number of subjects a DEG needs to correctly classify to be considered a good predictor.

**Step 3** consists in the analysis of the binary matrix ${ℳ}_{P\times N}$, where each row ${ℳ}_{\left(p,\cdot \right)}$ is represented by one of the *P* binary vectors $\hat{y}$ created in step 2 and each column ${ℳ}_{\left(\cdot ,i\right)}$ contains the classification labels assigned to subject *i* by each predictor.

In an unrealistic situation where all the DEGs have *pp* = 1, meaning that ${\hat{y}}_{i}={\bar{y}}_{i}$, ∀i∈{1,…,N}, ℳ would have the first *n* columns filled with 0 and the remaining *m* columns filled with 1.

To select the peculiar predictors for ‘C0’, denoted by $G_{0}$, the algorithm focuses on the first *n* column of ℳ containing the *n* samples belonging to class 0. It detects the features that assign the correct class label to the majority of the samples and simultaneously detects the most mislclassified samples. In fact, the basic idea is that the peculiar genes are those capable to correctly classify a subject when the majority of genes misclassifies them.

The criterion used to identify the most misclassified subjects is based on the analysis of the *n* columns of ℳ. Whenever the p-th gene misclassifies the i-th sample then ${ℳ}_{\left(i,p\right)}=1$ since we are focusing on samples whose correct classification is 0. Hence, the sum overall the i-th column of ℳ represents the number of features misclassifying the sample. The column indexes corresponding to highest sum values are the most misclassified samples. The same procedure applies to samples belonging to class 1, being careful to minimize the sum values instead of maximizing them, in order to find the most misclassified samples. To formalize this procedure, *K*_*k*_, *k* ∈ {0, 1} indicates the chosen quantile of the misclassification distribution to detect the most misclassified subjects in the two classes:

$$\begin{array}{c} \sum_{i=1}^{n}M_{\left(\cdot ,i\right)}>K_{0},\;\sum_{l=n+1}^{N}M_{\left(\cdot ,l\right)}<K_{1}. \end{array}$$ (4)

The *peculiar genes* are now easily detectable as the genes (rows of ℳ) voting for the correct classification of the most misclassified subjects and they are denoted by $G_{0}$ and $G_{1}$ for ‘low responders’and ‘high responders’respectively.

It may occurs that either $G_{0}=\emptyset$ or $G_{1}=\emptyset$. In this case, one can relax the thresholds that define the misclassified samples or introduce a ‘tolerance’ in the features choice, for example including features misclassifying a pre-selected number of most misclassified samples.

The final selected signature will be the union of the two lists of features, denoted by

$$\begin{array}{c} S=G_{0}\cup G_{1} \end{array}$$ (5)

#### The classifier parameters setting

The PGS procedure provides a signature *S* that is used to build our classification model. Classification algorithms extract meaningful rules from available data to build a model capable of correctly classify new inputs with the right label. Learning procedures can be supervised or unsupervised. In the first case the problem is presented with example inputs and their desired outputs, given by *a priori* knowledge and the goal is to learn a general rule that maps inputs to outputs. In the second case no labels are given to the learning algorithm, leaving it on its own to find structures capable of classify its input [27, 28].

In this paper we present a supervised approach using the Support Vector Machine (SVM) as classifier [29]. SVMs are widely studied and used classifiers in many different domains as described in [30] and [31]. Recently, they also became a useful tool in the classification of samples coming from microarray experiments [32, 33].

SVMs separate a given set of binary labeled training data finding the equation of the hyperplane that maximizes the distance between the two classes. In case of noisy/sparse data the linear separation of the two classes is not always possible in the input space. In this case SVMs can perform a non-linear mapping of data in a so called *feature space* where the classes are linearly separable by using the ‘kernel’ technique [34].

Let S be a sample of *n* labeled data points: *S* = {(*x*^1^, *y*^1^), …, (*x*^*n*^, *y*^*n*^)}, where $x^{i}\in {\mathbb{R}}^{n}$, *y*^*i*^ ∈ {0, 1} and let $\phi :I\subseteq {\mathbb{R}}^{n}\to F\subseteq {\mathbb{R}}^{N}$ be a mapping from the input space to the feature space F. The kernel technique allow to define the inner product in the feature space without computing the mapping of inputs **x**^*i*^ → *ϕ*(**x**^*i*^) by the relation *K*(**x**^*i*^, **x**^*j*^) = *ϕ*(**x**^*i*^) ⋅ *ϕ*(**x**^*j*^). Classical choices for kernel functions are

$$\text{Gaussian}:K_{ij}({\text{x}}^{i},{\text{x}}^{j})=e^{-\frac{∥{\text{x}}^{i}-{\text{x}}^{j}∥}{\sigma^{2}}}$$

$$\text{Polynomial}:K_{ij}(x^{i},x^{j})={\left(\langle x^{i},x^{j}\rangle +c_{0}\right)}^{d}$$

$$\text{Sigmoid}:K_{ij}(x^{i},x^{j})=tanh(ax^{iT}x^{j}+r)$$

where *σ*, *d*, *a*, *r* are kernel parameters to be tuned.

SVMs give the possibility to chose a constant *c* to account penalties for misclassification. This pipeline uses SVM as classifier and explores a grid of parameters in order to detect the setting which allows the best classification performances on the selected metric.

In order to assess the accuracy of the model, it is common practice to split the original data set into *k* *training* and *validation* sets [35, 36]. This procedure is called *k-fold cross-validation* and usually uses the 80% of the original data for training the model and the 20% of the remaining samples to check the accuracy of the model on new inputs [37]. All the performances of the model presented in this work are evaluated with a 10 fold cross-validation procedure.

## Results

To evaluate the proposed approach, we applied the PGS on the two public microarray data sets described in Materials and Methods and we compared the classification performances to those obtained using MRMD software and the CMA package.

### Vaccination dataset

The proposed pipeline is specific for binary classification tasks. As described in Materials and Methods section, the binary classification of the samples was based on the 4-fold increase in the antibody titers levels: subjects reaching a 4-fold increase between day 1^−^ and day 28 against at least one of the three antigens were labeled as ‘high responders’while the others were labeled as ‘low responders’.

**Fig 2:**
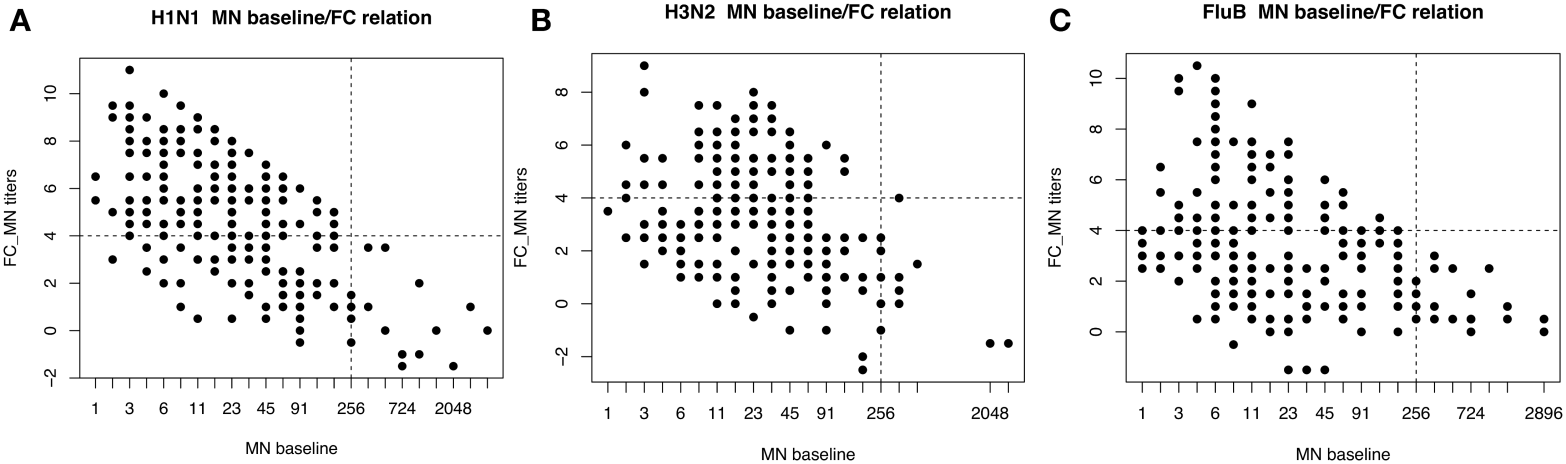
Negative association between baseline MN titers and titers fold-increase. Almost all subjects with a baseline MN titers higher than 256 did not reach the 4 fold-increase, fact that may lead to confounding effects in the classification procedure. This condition was met with all three antigens of the vaccine H1N1 (A), H3N2 (B), FluB(C).

Fig 2A, 2B and 2C show that subjects with antibody-titers *T* such that *T*_*i*_ > 256, *i* ∈ { H1N1, H3N2, FluB } at the baseline, never exceed a 4-fold increase. Therefore, to avoid misclassification due to pre-existing immunity, all subjects with a baseline titer *T*_*i*_ > 256 were excluded from subsequent analysis.

According to the classification criterion, 49 of the remaining samples were labeled as low responders and 144 as high responders. With such a relatively small sample size, it appears evident the imbalance between the two class sizes: the number of high responders is around three times the number of low responders. This situation is not surprising: the vaccination is expected to elicit a good response in the majority of the people.

#### The features selection procedure

PGS, as described in Materials and Methods, consists of three steps. The identification of DEGs, (**Step 1**), was performed using *limma* package [17], available under Bioconductor distribution. We set as contrasts the difference in genes expression one day before and one day after the vaccination. This step allowed us to reduce the data dimensionality of one magnitude order: from 28450 genes present on the microarray, only 3605 were significantly differentially expressed (Adjusted p-value ≤ 0.01).

**Fig 3:**
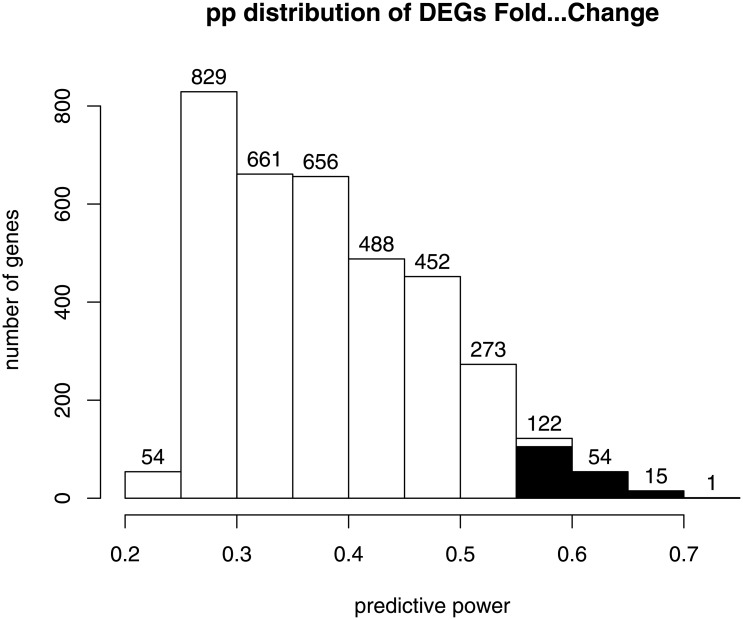
Histogram showing the *predictive power* distribution of all DEGs. The right 5th percentile of the distribution, in black, represents the number of genes selected as *good predictors*. The numbers on the columns of the histogram represent the number of genes with the same *pp*.

For the detection of the *good predictors* (**Step 2**), we applied the logistic regression model using fold-change of the gene expression between day 0 and day 1 as independent variable. To predict the class label of each subject we then applied the criterium presented in Eq (2), setting *τ* = 0.6. The fit of the model allowed us to compute, for each gene, the proportion of subjects correctly classified, which is referred to as *predictive power* (*pp*). We defined good predictors the genes whose *pp* belonged to the 5th percentile of the *pp* distribution of all the DEGs as showed in Fig 3.

The *peculiar genes* selection (**Step 3**), required the analysis of the binary matrix obtained in Step 2. Computational experiments showed that by setting *K*_0_ = 165 and *K*_1_ = 20, we identified 2 highly frequently misclassified ‘low responders’and 5 frequently misclassified ‘high responders’. The algorithm detected 11 *peculiar genes* belonging to $G_{0}$ and 19 belonging to $G_{1}$.

Tables 1 and 2 shows that the Gene Ontology (GO) on *S*, performed with Erichr [38, 39], found statistically significant enrichments in the biological processes involved in the immune response to virus. More specifically, the cytokine-mediated signaling pathway and in the type-I interferon signaling pathway are known to play an important role in host defense against virus. Table 3 shows that the antigen processing and presentation pathways are significantly enriched in the signature *S*, confirming the biological relevance of PGS.

**Table 1:** Biological process enriched in S.

Biological Process	Adjusted p-value
Defense response to virus	< 0.001
Defense response to other organisms	< 0.001
Cytokine-mediated signaling pathway	< 0.001
Regulation of immune effector process	< 0.001
Type I interferon signaling pathway	< 0.001

**Table 2:** Molecular functions enriched in S.

Molecular Functions	Adjusted p-value
MHC Protein Complex Binding	< 0.01
MHC Class II Protein Complex Binding	< 0.01

**Table 3:** Transcription factors enriched in S.

Transcriptions factors	Adjusted p-value
Antigen Processing and Presentation	< 0.01
Influenza A Homo Sapiens	0.01
Graft-versus-host disease Homo sapiens	0.01
Herpes Symplex Infection	0.01
Intestinal Immune Network for IgA production	0.01

**Fig 4:**
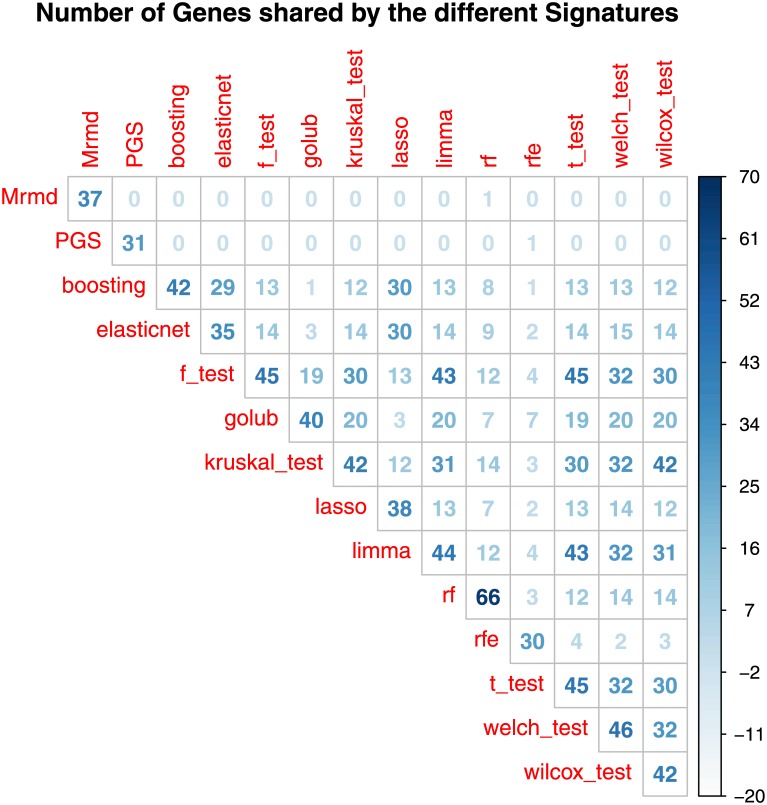
Number of genes shared by the signatures selected from the different feature selection methodologies.

To conclude the comparison of the feature selection procedures, we compared the lists of genes belonging to the different signatures. Fig 4 showed that MRMD, PGS and Random Forest selected signatures with the lowest overlap with all the others, whereas the other features selection procedures show a good overlap of genes.

To further investigate the properties of the genes shared among the different sigantures, we detected 43 genes selected by at least 5 different feature selection procedures. The GO perfermed on the list of the most selected genes showed results reported in Table 4, where no biological process is significantly enriched, suggesting that even considering the union of the most selected genes in the different signatures, the number of genes representing a particular biological process is too low to lead to a significant enriched pathway.

**Table 4:** Biological processes enriched in the ‘most selected genes’list.

Biologica Process	Adjusted p-value
Regulation of cellular extravasation	0.1
Regulation of cellular extravasation	0.1
Protein localization to vacuole	0.4
Protein localization to lysosome	0.4
Positive regulation of protein deacetylation	0.4

#### Classification results

We performed classification experiments using the feature selected as described in the previous paragraph to build a classification model with SVMs using a 10-fold cross-validation. Table 5 shows the results obtained applying our pipeline on the two most interesting cases for this set: the maximization of the accuracy on the entire validation set and the maximization of the specificity -i.e. when we want to detect all the samples belonging to the underrepresented class-.

**Table 5:** Transcription factors enriched in S.

Metric	Kernel	Degree	Coefficient	Cost	Class Weight	Accuracy
Overall Accuracy	Polynomial	5	1	0.1	$w_{C_{0}}=1$, $w_{C_{1}}=1$	82%
Sensitivity	Polynomial	6	-6	1000	$w_{C_{0}}=300$, $w_{C_{1}}=1$	1

The 10 fold-cross validation showed that the model reached its best performance maximizing the overall accuracy when the kernel of the SVM is a polynomial; -*i.e.* of the form *K*_*ij*_(**x**^*i*^, **x**^*j*^) = (〈**x**^*i*^, **x**^*j*^〉 + *c*_0_)^*d*^, where *d* = 5 is the degree and *c*_0_ the initial coefficient. The associated *cost* is 0.1, where the cost parameter for an SVM represents the tolerance of misclassification within each training example. When the parameter is small it means that the SVM looked for a larger-margin separating hyperplane allowing misclassification. To maximize the overall accuracy, the weight vector, the parameter accounting for the imbalance in the SVM has equal components: the same weight is given to both classes. Interestingly the situation is completely different when we want to detect all the subjects belonging to the underrepresented class: in this case, the imbalance in the classes size is reflected by the weight vector that shows the same strong imbalance between its two components.

Table 6 shows the best classification performances using different feature selection procedures in combination with the same classifier SVM where the tuning was possible only for fewer parameters. The list of genes selected by all the feature selection procedures tested are reported in S1 File.

**Table 6:** Accuracy on validation data set.

Feature Selection Method	Accuracy
**PGS**	**82** **%**
MRMD	75%
Random Forest	74%
F.test	69%
T.test	69%
Limma	68%
Welch	68%
Boosting	66%
Golub	66%
Kruskal Polynomial	66%
Wilcox	66%
Lasso	64%
Elastic Net	62%
Recursive Feature Elimination	59%

### Cancer data set

In Cancer data set description we pointed out that this data set is composed by paired-samples: each subject indeed, provided two samples of tissue, one normal and one tumor. This important remark has two main consequences on the subsequent analysis:

1. no risk of misclassification in labeling the samples with 0 or 1
2. perfect balance between the size of the two classes, with 60 samples in each.

#### The features selection procedure

**Fig 5:**
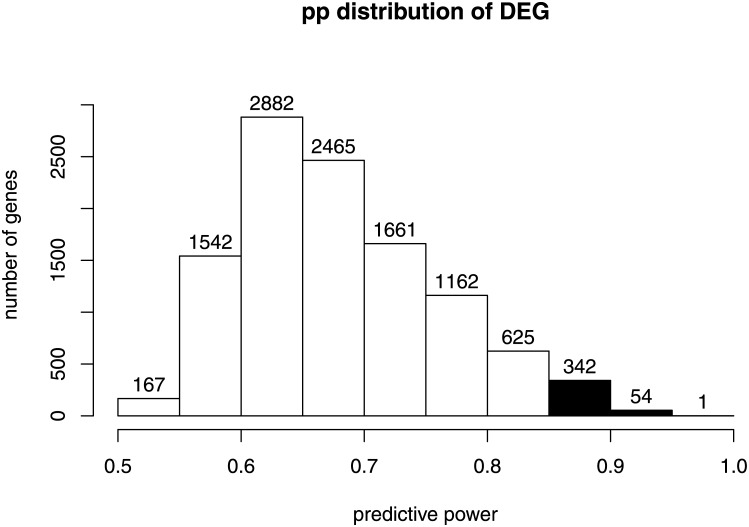
Histogram showing the *predictive power* distribution of all DEGs. The 99th percentile of the distribution, in black, represents the number of genes selected as *good predictors*.

To detect DEGs in this case study (**Step 1**) we used the *limma* setting as contrast the class labels of the samples. We detect 10901 DEGs starting from a chip containing 21655 genes. To detect the *good predictors*, as described in **Step 2**, we applied the logistic regression model using the gene expression of each sample to predict its class. For this dataset the *τ* used to predict the class labels of the samples was *τ* = 0.5. We obtained the *pp* distribution and, we defined good predictors the genes whose *pp* belonged to the 99th percentile of the *pp* distribution of all the DEGs as showed in Fig 5.

For this data set, the peculiar gene selection (**Step 3** of our procedure) required the analysis of the binary matrix obtained in Step 2. Computational experiments showed that by setting *K*_0_ = 10 and *K*_1_ = 80 this analysis led to the detection of 2 highly frequently misclassified ‘low responders’ and 6 highly frequently misclassified ‘high responders’. The algorithm, found 4 *peculiar genes* belonging to $G_{0}$ and 20 *peculiar genes* belonging to $G_{1}$. The GO performed on the signature S did not show any biological process nor molecular function significantly enriched whereas the analysis using the oncogenic signature database showed an enrichment on epidermal growth factor receptors as reported in Table 7.

**Table 7:** GO using oncogenic signature database.

Oncogenic Signature	Adjusted p-value
Epidermal Growth Factor Receptor (EGFR)	0.007
Kirsten rat sarcoma viral oncogene (KRAS)	0.01

**Fig 6:**
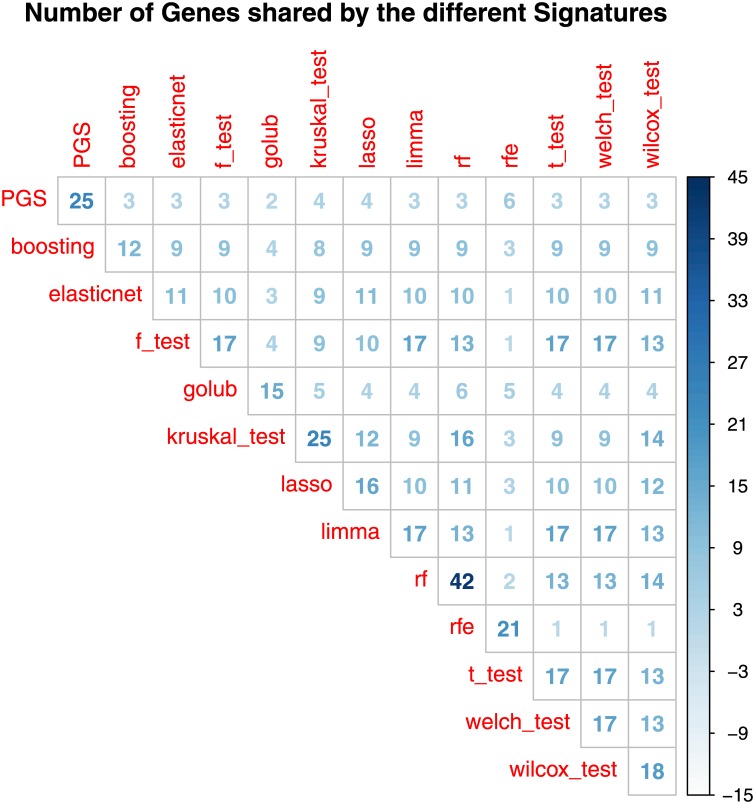
Number of genes shared by the signatures selected from the different feature selection methodologies.

Also for this data set, we compared the lists of genes selected by the different feature selection procedures. As in the vaccination data set, Fig 6 shows that the overlap among the genes selected by PGS and MRMD with those selected by the other feature selection procedures is low. On the contrary, despite the small number of genes per signature, the other feature selection methods seem to have a better agreement on the choice of the predictors. The list of genes selected by all the feature selection procedures tested are reported in S2 File.

Accordingly to what we did for the vaccination data set, we selected the genes that appeared in at least 5 different signatures as the ‘most selected genes’and we performed a gene ontology study. The GO results showed that no biological process nor molecular functions were significantly enriched, results confirmed also for the ‘most selected genes’. However, using the oncogenic signature database, we found again a significant enrichment for the epidermal growth factor, p-value <0.05 but not for KRAS. Significant enrichments were detected for genes up-regulated during early stages of differentiation of embryoid bodies from embryonic stem cells or embryonic fibroblasts (ESC V6.5 UP EARLY.V1 UP, NFE2L2.V2, PRC2 SUZ12 UP.V1 UP, ESC V6.5 UP LATE.V1 UP). The results of GO performed on the oncogenic database are showed in Table 8.

**Table 8:** GO using oncogenic signature database.

Oncogenic Signature	Adjusted p-value
ESC V6.5 UP EARLY.V1 UP	0.046
NFE2L2.V2	0.046
EGFR	0.046
PRC2 SUZ12 UP.V1 UP	0.046
ESC V6.5 UP LATE.V1 UP	0.046

#### Classification results

We applied PGS method to the cancer data set and compared the results with those obtained by using the CMA package with all the feature selection methods available. For this data set no parameter tuning was necessary: the optimal results were obtained by using the default settings of the SVM classifier, choosing a polynomial kernel: the default degree is 3, the initial coefficient is 1. The optimal cost here is, again, 0.1. The perfect balance between the two classes size lead to the logical choice of a weight vector whose components are equal (*i.e.* *w*_0_ = *w*_1_ = 1).

Table 9 shows the mean results obtained applying our classification method with a 10-fold cross-validation, whereas Table 10 shows the CMA performances.

**Table 9:** Transcription factors enriched in S.

Metric	Kernel	Degree	Coefficient	Cost	Class Weight	Accuracy
Overall Accuracy	Polynomial	3	1	0.1	$w_{C_{0}}=1$, $w_{C_{1}}=1$	98.5%
Sensitivity	Polynomial	3	1	0.1	$w_{C_{0}}=1$, $w_{C_{1}}=1$	1

**Table 10:** Accuracy on validation data set.

Feature Selection Method	Accuracy
**PGS**	**98.5%**
Boosting	99%
Elastic Net	99%
F.test	99%
Golub	99%
Kruskal Test	99%
Lasso	99%
Limma	99%
Random Forest	99%
Recursive Feature Elimination	99%
T.test	99%
Welch	99%
Wilcox	99%
MRMD	NA

The ‘NA’for the MRMD method means that the machine run out of memory for this data set.

## Discussion

The advent of HT technologies are fostering the implementation of different computational approaches for classification tasks. However, intrinsic characteristics of the data set, such the imbalance between the size of the classes, still represent an issue for the classification purpose.

In this paper we present a new feature selection method in 3 steps, called Peculiar Genes Selection, for the analysis of high dimensional data sets. The proposed pipeline detects the features that characterize the two classes and use them as a biomarker for predicting the class label of new inputs.

We applied PGS on two different data sets and then compared the classification performances with those obtained using other features selection methods already implemented in the CMA package and MRMD software. Following the recent literature about classification tasks with biological data, we decided to use as classifier an SVM. However, the presented pipeline can be used in combination with any other classifier that better suits the researcher purposes.

Two case study are considered, both concerning microarray experiments: one from a vaccination trial, the other from a cancer study. Despite all data come from microarray experiments, the two data sets have different characteristics that impact on the classification task. In the vaccination case, the blood samples come from healthy subjects who probably already encountered influenza virus before being enrolled for the trial; this situation is clearly reflected by the baseline effect underlined in Results section. Additionally, the vaccination data set required a first step of analysis to assign the class label to samples, step that was unnecessary for the cancer data set. In such a scenario we have to consider also the noise present in the data, explained by the across-subjects variability and by the fact that the expression of transcripts in the whole blood is a surrogate tissue to measure the immune response.

Second, the strong imbalance in the two classes size prevent the correct classification of both classes simultaneously: either the SVM privileges the overall accuracy penalizing the underrepresented class or it can detect all the underrepresented class but does not correctly classify the other one.

For this case study, the classification results reported in Tables 5 and 6 show that it is not possible to reach an overall accuracy higher than 82% using PGS as feature selection method and an averaged overall accuracy of 66.4% using the features selection methods included in the CMA package. PGS detected a signature of 14 genes belonging to $G_{0}$ and 17 genes belonging to $G_{1}$. The GO of the resulting signature S showed enrichments in response to virus and in cytokines-mediating signaling pathways, confirming the biological meaning of the proposed procedure.

In the cancer case-study PGS detected a signature of 40 genes, 27 in $G_{0}$ and 13 in $G_{0}$, whose GO on an Oncogenic Signature database shows enrichment in epidermal growth factor receptors accordingly to recent literature [40]. The biological processes enriched are related to DNA replication processes in sprouting angiogenesis, but they are not associated with significant adjusted p-values, see S3 File.

In the conclusions reported in [22], the authors underline the significant role of the axons signaling pathway in the survival analysis; interestingly, we found an enrichment in the axon guidance signaling pathway as well, but it wass not associated with a significant adjusted p-value.

The overall accuracy in this case is around 99% for all the feature selection methods tested, result that can be explained with the absolute absence of imbalance in the experimental design along with the fact that the gene expression was taken from normal and tumor tissues, in other words it is a direct measure.

## Conclusion

Microarray experiments measuring gene expression levels are source of important biological informations. Machine-learning algorithms are used to build predictive models and to find biomarker for classification tasks from data sets coming from microarray experiments. The high dimensionality of these technology outputs requires a first step of dimensionality reduction and the necessity of not losing important information gave rise to lots of feature selection approaches.

However, the characteristics of the data set analyzed, such as the biological conditions tested, the source of the genetic material and the human gene expression variability, still have a strong impact on the algorithms affecting their classification performances. When the samples are taken from direct sites of the considered conditions, the available computational approaches are capable of shrink the information contained in the microarray in a small set of genes and detect a biomarker as proved in the cancer related case-study. In vaccine-related studies we need to be more careful and deal with the fact that people undergoing vaccination are healthy, condition that makes difficult to detect a real significant gene expression change. The proposed procedure improve the classification performances in case of imbalanced data sets by selecting genes that are predictive for the two classes separately, reducing the risk of a loss of information about the underrepresented class when compared to other feature selection methods.

## Supporting information

S1 File Lists of genes selected by the different feature selection procedures for the vaccine data sets. (XLSX)

S2 File Lists of genes selected by the different feature selection procedures for the cancer data sets. (XLSX)

S3 FileBiological Processes enriched on S in Cancer study.(XLSX)
